# Vallecular varicose lesion: A rare etiology for upper aerodigestive tract hemorrhage

**DOI:** 10.1016/j.bjorl.2023.101330

**Published:** 2023-09-18

**Authors:** Elaine Costa, Vinícius Domene, Yuri Costa Farago Fernandes, Thiago Pires Brito, Graziela de Oliveira Semenzati, Agrício Nubiato Crespo

**Affiliations:** a Faculdade de Ciências Médicas da Universidade Estadual de Campinas, Campinas, SP, Brazil; b Departamento de Otorrinolaringologia, Cirurgia de Cabeça e Pescoço, Universidade Estadual de Campinas (UNICAMP), Campinas, SP, Brazil

## Introduction

Oral cavity hemorrhage primarily originates from either the upper airway or the Gastrointestinal (GI) tract. Varicose lesions account for a rare cause of bleeding in the hypopharynx, complicating clinical evaluation due to difficulties in discerning the source of bleeding. We report a case of severe hemorrhage resulting from a varicose lesion located in the vallecula. It is critical to consider such lesions when investigating hematemesis of unknown origin. A literature review was carried out to evaluate related cases, clinical approaches, and surgical management. Awareness of this condition contributes to accurate diagnosis and avoids unnecessary invasive diagnostic procedures.

## Case report

An 85-year-old male patient with a history of treated hepatitis C, non-cirrhotic, presented to the Emergency Department (ED) with a three-day history of melena associated with daily episodes of oral cavity bleeding during and after eating, not associated with coughing or vomiting. The patient denied using anticoagulants or taking nonsteroidal anti-inflammatory drugs. An earlier endoscopy revealed no varicose veins in the esophagus and a liver of normal size and blunt edges.

**Figure 1:**
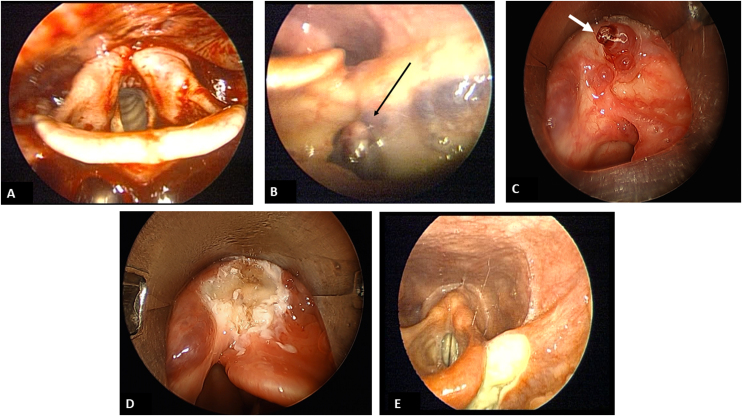
(A) Indirect laryngoscopy showing a large amount of fresh blood in the hypopharynx. (B) Indirect laryngoscopy revealing varicose vessels, black arrow, in the left vallecula. (C) Direct laryngoscopy with umbilicated varix, white arrow, with clot adhered to the apex. (D) Direct laryngoscopy shows the vallecula’s left region after cauterization of the lesion. (E) Indirect laryngoscopy on the 8th post-operative day evidencing granulation tissue.

Upon examination, the patient was hemodynamically stable with no signs of active bleeding, chronic liver disease, or head and neck lesions. Initial laboratory results in the ED revealed a hemoglobin level of 10.7 g/dL, hematocrit of 31.7%, platelet count of 196,000 and normal coagulation and leukocyte count. A neck and chest Computed Tomography (CT) scan did not reveal any tumor or vascular malformations. Three days post-admission, the patient’s hemoglobin levels fell from 10.7 g/dL to 7.4 g/dL and hematocrit from 31.7% to 23%. Hemoglobin and hematocrit were monitored every six hours. Upper gastrointestinal endoscopy was inconclusive, prompting a consultation with the otolaryngology department due to suspected upper aerodigestive hemorrhage. Initial indirect laryngoscopy revealed a substantial amount of fresh blood in the hypopharynx, but no lesions were initially detected (Fig. 1A).

A second upper gastrointestinal endoscopy showed a clot in the left vallecula, which was actively bleeding upon removal. The patient underwent reevaluation by the otolaryngology department. Indirect laryngoscopy revealed an exophytic angiomatous lesion on the lateral pharyngeal wall, extending to the left vallecula (Fig. 1B), suspected to be a vallecula varix. A neck angiotomography was performed, with no evidence of mass lesion or anomalous arterial or venous circulation.

**Figure 2:**
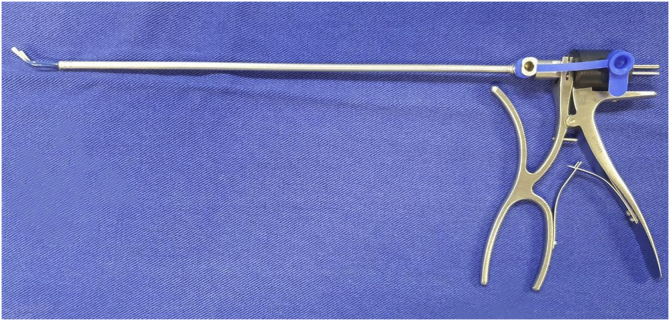
Micro laryngeal bipolar forceps.

Direct laryngoscopy was then performed with endoscopic laryngeal microsurgery (Supplementary Material Video 1). A varix with an umbilicated surface and an adhered clot was identified on the left vallecula, and successful diathermy using micro laryngeal bipolar forceps was performed successfully (Fig. 2 and Figs. 1C and 1D). The patient received a total of two units of packed red blood cells during hospitalization due to a drop in hemoglobin levels below 7 g/dL prior to surgical intervention. No recurrence of bleeding was observed at follow-up (Fig. 1E), suggesting that the varicosities were indeed the source of bleeding.

## Discussion

Common presentations of upper aerodigestive tract hemorrhage include hematemesis and melena, and potential etiologies encompass peptic ulcer, varicose veins, esophagitis, gastritis, duodenitis, Mallory-Weiss laceration, and tumors. Vallecula bleeding can occur due to benign or malignant tumors, traumatic intubation, congenital malformations, blood dyscrasia, or in association with portal hypertension or cirrhosis.^1^, ^2^

The venous plexus, a tributary of the internal jugular vein or brachiocephalic veins, drains the pharyngeal and laryngeal structures.^3^ This pattern of venous drainage could contribute to the formation of pharyngeal varicose veins.

Vallecula varicosity represents an uncommon cause of upper aerodigestive bleeding with few documented cases. To the best of our knowledge, this case is the first reported in Brazil and the 11th in the English-language literature. Due to their location, small size, and intermittent bleeding, such varicosities are often difficult to identify. They can present as hemoptysis, hematemesis, epistaxis, and/or melena and may lead to high morbidity due to moderate to severe bleeding and resulting hemodynamic repercussions.^2^, ^4^, ^5^

Treatment options include sclerotherapy, carbon dioxide laser ablation, and thermal cauterization (micro laryngeal bipolar forceps).^1^, ^3^, ^4^ In this case report, we utilized micro laryngeal bipolar forceps, enabling rapid and straightforward cauterization.

Thus, rare conditions like vallecular or pharyngeal varix should not be overlooked as delay in diagnosis can be life-threatening. A meticulous evaluation of the vallecula and hypopharynx through endoscopic examination is essential for early identification of the bleeding source and adequate definitive hemostasis.

## Conclusion

When assessing undetermined-origin hematemesis, varicose lesions in the vallecula warrant consideration and investigation. Surgical treatment demonstrates promising results, as evidenced both in the literature and in our case. Micro laryngeal bipolar forceps prove to be a valuable tool for managing such situations.

## Funding

The authors have no funding or financial relationships.

## Conflicts of interest

The authors declare no conflicts of interest.
