# Supplementary material for: Vallecular varicose lesion: A rare etiology for upper aerodigestive tract hemorrhage
Source: Braz J Otorhinolaryngol. 2023 Sep 18;89(6):101330. doi: 10.1016/j.bjorl.2023.101330 (PMC10520730; doi:10.1016/j.bjorl.2023.101330)
Supplement: Supplementary file 1 [file mmc1.docx]

BJORL-D-23-00087_ Supplementary Material

**Video 1** Direct laryngoscopy with the performance of vallecular varix cauterization through endoscopic laryngeal microsurgery. This video shows surgical steps and the use of micro laryngeal bipolar forceps.

**Alt text:** This video describes surgery steps: Inspection through direct laryngoscopy with 0° rigid optics shows a varix with an umbilicated surface and adhered clot identified on the left vallecula. Palpation of the lesion using a cotton ball and alligator forceps. After, we performed diathermy using micro laryngeal bipolar forceps successfully. The total procedure duration was less than 10 minutes.
